# Effectiveness of Biological Approaches for Removing Persistent Organic Pollutants from Wastewater: A Mini-Review

**DOI:** 10.3390/microorganisms12081632

**Published:** 2024-08-09

**Authors:** Carmen Mateescu, Eduard-Marius Lungulescu, Nicoleta-Oana Nicula

**Affiliations:** National Institute for Research and Development in Electrical Engineering ICPE-CA, 313 Splaiul Unirii, 030138 Bucharest, Romania; carmen.mateescu@icpe-ca.ro (C.M.); nicoleta.nicula@icpe-ca.ro (N.-O.N.)

**Keywords:** persistent organic pollutants, environment, wastewater, bioremediation, bacteria, fungi

## Abstract

Persistent organic pollutants (POPs), including organochlorine pesticides, polycyclic aromatic hydrocarbons, polychlorinated biphenyls, polychlorinated dibenzo-p-dioxins, and polychlorinated dibenzo-p-furans, pose significant hazards to the environment and living organisms. This concise review aims to consolidate knowledge on the biological processes involved in removing POPs from wastewater, an area less explored compared to conventional physico-chemical methods. The focus is on the potential of various aerobic and anaerobic microorganisms, fungi, and bacteria for efficient bioremediation, mitigating or eradicating the deleterious effects of these chemicals. The review scrutinizes individual bacterial strains and mixed cultures engaged in breaking down persistent organic pollutants in water, highlighting promising results from laboratory investigations that could be scaled for practical applications. The review concludes by underscoring the opportunities for exploring and advancing more sophisticated bioremediation techniques and optimized bioreactors. The ultimate goal is to enhance the efficiency of microbial-based strategies, implicitly reducing the environmental impact of persistent chemicals.

## 1. Introduction

The rapid expansion of industrialization and urbanization in the past century has led to a significant upsurge in chemical consumption, becoming indispensable not only in numerous industries but also in agricultural practices, particularly for soil fertilization. The widespread use of certain chemicals during the 1950s–1970s, pivotal for their role in various applications, has left a lasting environmental impact. These chemicals (i.e., polychlorinated biphenyls—PCBs; dichlorodiphenyltrichloroethane—DDT; hexachlorobenzene—HCB; etc.), now recognized as persistent organic pollutants (POPs), exhibit remarkable toxicity and continue to endure in the environment, posing ongoing threats to ecosystems and human health [1].

Article 6 of the Stockholm Convention on Persistent Organic Pollutants (POPs) adopted in 2001 requests parties to “take appropriate measures so that wastes consisting of, containing or contaminated with persistent organic pollutants are disposed of in such a way that the POP content is destroyed or irreversibly transformed so that they do not exhibit the characteristics of POPs or otherwise disposed of in an environmentally sound manner” [2].

POPs primarily consist of halogenated organic compounds that exhibit high resistance to most environmental degradation processes. Their elevated chemical stability stems from the non-reactivity of the carbon–chlorine (C–Cl) bonds to hydrolysis and photolytic degradation. The strength of the C–Cl bond is attributed to its strong polarization, where the electron cloud is concentrated on the halogen, acquiring a positive charge, while carbon experiences an electron deficit, resulting in a positive charge as well. This disparity in electric charge gives rise to a robust carbon–halogen bond, characterized by a compelling attraction. Moreover, the organic molecule forms shorter and stronger bonds when it contains a greater number of halogen atoms linked to the same carbon atom [3,4].

Despite their notable chemical stability, certain molecular modifications in the environment do not necessarily result in simpler and less toxic compounds; in fact, some descendants degradation products of persistent organic pollutants can be as complex and even more toxic than their parent molecules, as noted by Zacharia [5]. The eradication of POPs from contaminated soils or discarded materials and components typically involves two fundamental technologies: combustion technology and non-combustion technology. High-temperature incineration technologies have been extensively employed for treating POP-contaminated soils and materials, effectively handling large volumes of various POP types. While incineration has shown satisfactory results, concerns persist regarding the potential environmental and health impacts associated with this method. The combustion of POPs can generate highly toxic by-products, such as polychlorinated dibenzo-p-dioxins (PCDDs) and polychlorinated dibenzo-p-furans (PCDFs), both established carcinogens.

Physical methods such as adsorption using activated carbon are commonly employed due to their high efficiency in capturing a wide variety of pollutants; its effectiveness is limited by carbon’s capacity and the need for regeneration [6]. Chemical methods like advanced oxidation processes (AOPs) offer an alternative [7,8,9]. AOPs, including ozonation and Fenton’s reaction, generate highly reactive molecules that break down persistent organic pollutants (POPs) completely, but require precise control and can be expensive due to high energy demands or costly chemicals [7]. Recognizing these drawbacks and the necessity for more cost-effective solutions has led to a growing interest in exploring and testing non-incineration technologies for the efficient removal of POPs from the environment [10].

Although the bulk of research into the biological degradation of persistent organic pollutants (POPs) has centered on soils contaminated with pesticides, as well as materials harboring polychlorinated biphenyls (PCBs) and polycyclic aromatic hydrocarbons (PAHs), there is a pressing need to investigate and synthesize existing knowledge on the elimination of POPs from wastewater. This specific area appears to be relatively underrepresented in the current body of literature, warranting focused attention and investigation to enhance our understanding of the biological processes involved in POP removal from wastewater environments.

Wastewater treatment is a critical component of modern environmental stewardship, essential for preserving the health of aquatic ecosystems and safeguarding human well-being. Traditional wastewater treatment methods, which predominantly rely on physico-chemical processes, are effective in removing conventional pollutants. However, these methods are often inadequate for the complete removal of POPs. Their persistence and complex chemical structures challenge the capabilities of conventional treatment plants, making the need for alternative approaches more pressing [6].

Biological techniques, which rely on the metabolic activity of microorganisms like bacteria and fungi, have emerged as promising alternatives for the removal of POPs from wastewater. These approaches harness the natural abilities of microorganisms to break down and transform persistent compounds by intracellular biochemical degradation into less harmful chemicals. Consequently, assessing the effectiveness of biological methods in eliminating POPs from the environment is a subject of considerable scientific interest [11].

This mini-review aims to explore and evaluate the various biological approaches used in the removal of POPs from wastewater. Through an in-depth analysis of the existing literature, we aim to shed light on the effectiveness of microbial-based methods, their advantages, their limitations, and their potential contribution to more sustainable and environmentally friendly wastewater treatment practices. Understanding the role of biological approaches in tackling POP contamination is essential for mitigating the risks associated with the persistent pollutants and advancing the field of wastewater treatment towards improved environmental and public health outcomes.

## 2. Methodology and Results

The selection of published papers to be used for this investigation was carried out using PRISMA recommendations [12]. The research methodology involved the following aspects, according to the PICO strategy:

1. ***Problem* (P)**: Challenges associated with the removal of persistent organic pollutants (POPs) using conventional treatment methods;
2. ***Intervention* (I)**: Biological treatment methods to effectively remove POPs from wastewater;
3. ***Comparison* (C)**: Conventional physicochemical treatment methods;
4. ***Outcome* (O)**: Outcomes in terms of POP concentration reduction, breakdown product formation, cost-effectiveness, and environmental impacts associated with biological POP removal.

This study aimed to address the following PICO question: “Are biological treatment methods effective in removing persistent organic pollutants (POPs) from wastewater”? The criteria for inclusion were as follows: research articles from 2017 onwards with full-text access, published in English, containing terms like “biological removal”, “bioremediation”, and “water pollution”, and providing novel insights relevant to the review. The exclusion criteria were as follows: articles published before 2017, book chapters, books, reviews, conference materials, notes, letters, short surveys, errata, retracted papers, editorials, non-English articles, and articles without substantial content on “biological removal of POPs” or “wastewater”.

Exploration of the literature was conducted using the SCOPUS database, chosen for its comprehensiveness, with the primary search term being “biological removal of POPs”. Subsequent article selection was automated, adhering to the predefined inclusion/exclusion criteria detailed earlier. Inclusion in the current review was determined subsequent to a thorough examination involving the comprehensive reading of the manuscripts.

Following the rigorous application of the aforementioned inclusion and exclusion criteria, and through a meticulous scrutiny of titles, abstracts, and full-text content, we pinpointed a total of 30 articles deemed appropriate for inclusion in this review.

**Figure 1:**
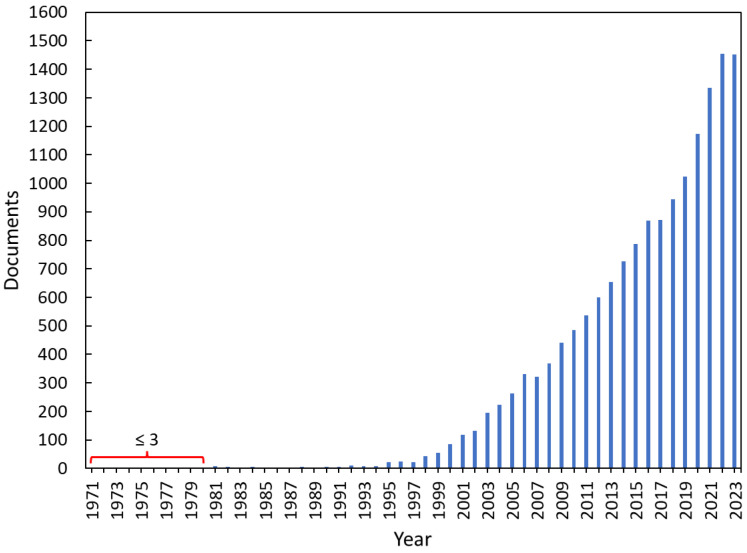
Number of documents from Scopus database related to “Persistent Organic Pollutants” in the keyword search.

The exploration of the Scopus database commenced with an initial keyword search for “Persistent Organic Pollutants”, yielding a total of 15,393 papers (Figure 1). The observed exponential increase in documents addressing POP pollution since 1995 is likely due to a confluence of factors: growing awareness of the severe environmental and health risks posed by persistent organic pollutants, increased scientific research and regulatory efforts to understand and mitigate these pollutants, and the development of more sophisticated analytical techniques to detect and quantify POPs in various environmental matrices.

Through the automated application of exclusion criteria, this extensive pool was refined to 5889 papers. Subsequent application of inclusion criteria further narrowed down the selection to 250 papers. A meticulous manual review, involving exclusion criteria (1–5), led to the examination of 156 papers at the title level, followed by scrutiny towards 110 papers at the abstract level and 88 papers at the full-text level. Ultimately, 30 papers, aligning with the predefined inclusion criteria, were selected for inclusion in this mini-review. These chosen articles collectively explore the domain of biological removal of persistent organic pollutants (POPs) from wastewater. To enhance the comprehensive contextual framework, we augmented our selection with pertinent works identified through a rigorous manual curation process within the SCOPUS database, employing targeted keyword-driven searches.

The selection of the relevant articles was carried out meticulously, considering that a large part of the documentary materials were mainly focused on the harmful health effects of POPs, on the sources of contamination and on the method of POP diffusion in the environment, as well as on POPs in soil and residues found in sediments, but very few published articles referred to their presence in wastewater. Another challenge in collecting relevant articles was that many articles addressed the conventional thermo-physico-chemical methods of eliminating POPs from the environment (e.g., incineration, coagulation, flocculation, adsorption, advanced oxidation) in particular, which face well-known disadvantages, such as high processing costs, unsatisfactory removal efficiency, the need for subsequent removal of used adsorbents, and the thermal transformation of some POPs such as polychlorinated biphenyls into unwanted dioxins and furans. Only relatively few articles addressed the microbiological decontamination of wastewater containing POPs; hence, we can state that research in this direction is in the early stages of development.

The generation of three-dimensional chemical structures was accomplished using Avogadro 1.2.0 software [13].

## 3. Microorganisms Employed for Removing POPs from Wastewaters

**Figure 2:**
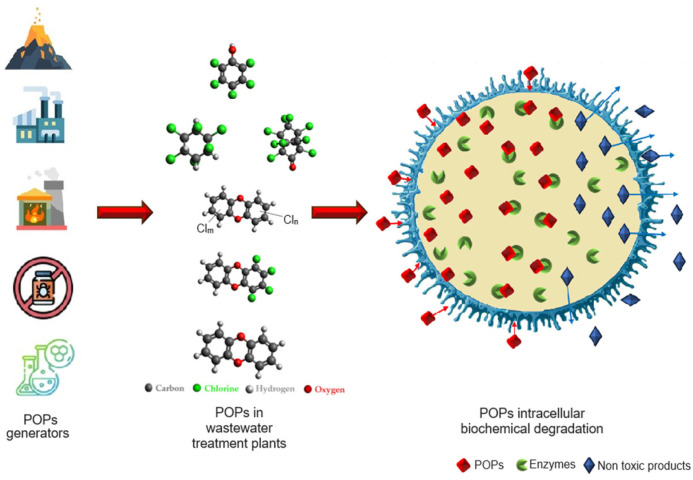
Schematic representation of the biodegradation mechanism of POPs.

Bioremediation represents a dynamic technique harnessing the activities of microorganisms, including both aerobic and anaerobic fungi and bacteria, for the purpose of decontaminating, destroying, or transforming hazardous substances into compounds that are either harmless or less dangerous (Figure 2).

The key players in this process are microorganisms, which exhibit ubiquity due to their widespread presence and isolation in diverse environments, spanning from animals, plants, and soil to water. The versatile nature of these microbes allows them to adapt and thrive in various conditions, making them effective agents in the remediation of contaminated sites. From soil and water to the complex worlds within plants and animals, these microorganisms play a crucial role in mitigating environmental pollution by facilitating the conversion of harmful substances into forms that pose reduced risks to ecosystems and human health [14,15,16,17].

### 3.1. Bacteria-Assisted Biodegradation of POPs from Wastewaters

**Figure 3:**
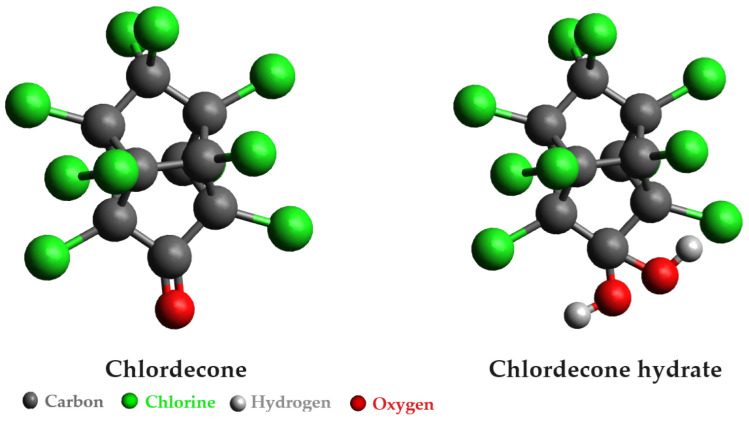
Structure of chlordecone and its hydrated form.

Various physical and chemical approaches have been employed to address the remediation of pesticides in water, with adsorption on activated carbon being a prevalent method. However, there has been a notable surge in interest in bioremediation techniques utilizing bacteria. Among the persistent organic pollutants drawing considerable attention from researchers is chlordecone, an organochlorine insecticide and fungicide extensively used from the 1960s to the 1990s and still prevalent in water and the food chain. Also known by the brand name Kepone, chlordecone poses significant health risks, acting as a reproductive and neurological toxicant, a carcinogen, and an endocrine-disrupting chemical. It forms bonds with liver proteins and plasma proteins, notably albumin and high-density lipoproteins [18]. The chlordecone molecule consists of a bishomocubane cage, ten chlorine atoms, and a ketone function. Notably, the ketone group undergoes spontaneous hydration, resulting in a stable gem-diol form recognized as chlordecone-hydrate (Figure 3). The distinctive structure of chlordecone imparts strong hydrophobic properties, fostering a robust affinity for organic matter. Additionally, this compound exhibits both acute and chronic toxicity, as highlighted by Della-Negra et al. [19].

In a study conducted by Amba Esegni et al. [20], strains of *Bacillus* sp. were isolated and assessed for their capability to metabolize chlordecone, serving as the sole carbon source in the experiment. The biodegradation of chlordecone was substantiated by the observed release and quantification of free chlorine, reaching its peak 10 days after the initiation of the experiment. This represented a dechlorination level equivalent to 19.5% of the total chlorine content present in the pesticide compound.

**Figure 4:**
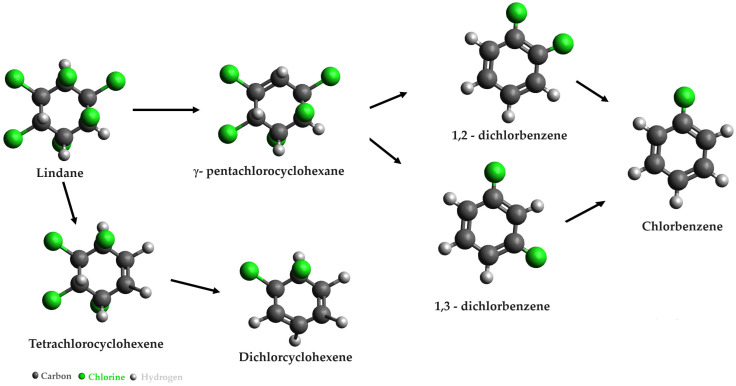
Anaerobic degradation pathways of lindane [22].

In the degradation experiments conducted by Barbance et al. [21], *Citrobacter* sp. 86 strains were employed for the breakdown of γ-hexachlorocyclohexane, commonly known as lindane. These experiments involved anoxic microbial incubations carried out in a glove box under daylight conditions. The monitoring of lindane degradation was conducted using gas chromatography–mass spectrometry coupled with headspace trap (HS-GC-MS) analysis. The anaerobic degradation pathways of lindane are illustrated in Figure 4 [22].

Bashir et al. [23] investigated the biotransformation of hexachlorocyclohexane (HCH) isomers using two *Dehalococcoides mccartyi* strains (195 and BTF08). The transformation of HCH isomers was monitored by analyzing potential metabolites or end-products, including tri-, di-, mono-chlorobenzene, and benzene, employing gas chromatography. Strains 195 and BTF08 exhibited the capability to transform not only γ-HCH and α-HCH but also β-HCH and δ-HCH. The primary observed metabolites were tetrachlorocyclohexene, with the end products identified as monochlorobenzene (MCB) and benzene.

Additionally, certain studies have demonstrated the degradation of HCH isomers under aerobic conditions. Aerobic HCH-degrading bacteria, identified thus far to belong to the family *Sphingomonadaceae*, are classified into three distinct groups: *Sphingobium japonicum* UT26, *Sphingobium indicum* B90A, and *Sphingobium francense* Sp+ [24].

An effective bacterial mixture able to degrade the mixture of PAHs (naphthalene, phenanthrene, fluoranthene and pyrene) was developed by Patel et al. [25] from polluted marine sediments. The four morphologically different bacteria were isolated from the bacterial species *Pseudomonas* sp., *Achromobacter* sp., and *Chelatococcus* sp. Among these species, only *Chelatococcus* sp. was not associated with PAH degradation before this study.

Arulazhagan et al. (2010) [26] employed a consortium of three halotolerant bacterial strains (*Ochrobactrum* sp., *Enterobacter cloacae*, and *Stenotrophomonas maltophilia)* for the degradation of PAHs. The bacterial consortium demonstrated significant efficacy, degrading over 95% of PAHs at various concentrations (5, 10, 20, 50, and 100 ppm) within a four-day period when exposed to a sodium chloride concentration of 30 g/L. However, at a higher sodium chloride concentration of 60 g/L, the degradation of different polycyclic hydrocarbons ranged from 39% to 45%.

Polychlorinated biphenyls (PCBs), comprising a complex mixture of 60–90 congeners, were extensively utilized in industrial applications during the 1930s and 1940s due to their exceptional physical and chemical properties. Despite their toxicity, PCBs persist in the environment because natural aquatic and soil biota struggle to metabolize them at a significant rate. The high chlorination of PCBs renders them highly insoluble in water, contributing to their resistance to biodegradation, as the compound’s water solubility plays a pivotal role in structural degradation [27].

Horváthová et al. [28] implemented an innovative strategy for PCB biodegradation by constructing binomial and trinomial consortia comprising four bacterial strains isolated from contaminated sediment. Significantly, the most successful consortia showcased the presence of the *Rhodococcus ruber* strain in various pairings: *R. ruber* and *A. xylosoxidans* (RA), *R. ruber* and *S. maltophilia* (RS), and *O. anthropi*, *R. ruber* and *A. xylosoxidans* (ORA). Findings indicated that the trinomial consortium ORA displayed diminished effectiveness compared to the binomial ones, exhibiting only a marginal 2% increase in biodegradation compared to the most efficient single strain. Both binomial consortia exhibited notable efficiency at a biomass ratio of 1:1, achieving approximately 80% biodegradation of the total sum of PCB congeners. Under these conditions, consortia outperformed individual strains in PCB biodegradation. Furthermore, the combination of *R. ruber* and *S. maltophilia* effectively eliminated highly chlorinated PCB congeners characterized by low bioavailability and high toxicity.

The aerobic and anaerobic biodegradations of PCBs have been reviewed by many scientists. Under aerobic conditions, bacteria of the genus *Pseudomonas* have been investigated for the oxidative dechlorination or hydrolytic dehalogenation of PCBs. *Pseudomonas pseudoalcaligenes* and *Stenotrophomonas maltophilia* exhibited significant efficacy in the removal of biphenyl (up to 91%) and 2-chlorobiphenyl (up to 81%) from wastewater, as reported in reference [29]. However, in the presence of Cr (VI) ions, the removal efficiency slightly decreased, reaching 57% for biphenyl and 49% for 2-chlorobiphenyl. Despite this reduction, a notable positive outcome was observed, with a simultaneous reduction in Cr ions of up to 56%. Borja et al. [30] found that some anaerobes (e.g., *Desulfomonas* sp., *Dehalobacter* sp.) and facultative anaerobes (e.g., *Enterobacter* strains) were effectively used for PCB dechlorination.

**Figure 5:**
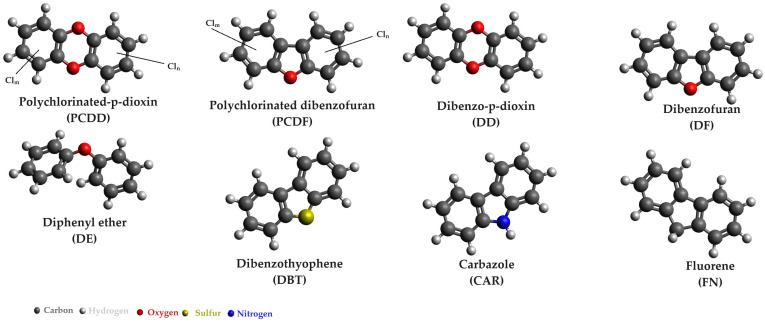
Chemical structure of chlorinated dioxins and related compounds.

Polychlorinated dibenzo-p-dioxins (PCDDs) and polychlorinated dibenzofurans (PCDFs) are a highly toxic organic compounds, and they can persist in the environment for over a century. Among them, 2,3,7,8-TCDD (tetrachlorodioxin) stands out as the most toxic congener. While less-chlorinated and unchlorinated analogues are comparatively less toxic than 2,3,7,8-TCDD, they are still considered part of this dioxin group, alongside halogenated biphenyl ethers, carbazole, fluorine, and their carboxylated or substituted derivatives. Figure 5 illustrates the chemical structures of some chlorinated dioxins and related aromatic compounds.

Research indicates that aerobic bacteria belonging to the genera *Sphingomonas*, *Pseudomonas*, and *Burkholderia* have the capacity to degrade less chlorinated dioxins. On the other hand, more chlorinated dioxins can undergo reductive dechlorination in anaerobic sediments, primarily facilitated by bacteria from the genus *Dehalococcoides*. This bifunctional microbial degradation process highlights the diverse strategies employed by different bacterial genera to address distinct chlorination levels in dioxins, demonstrating the potential for both aerobic and anaerobic pathways in the remediation of these environmental contaminants. Anaerobic sediments have demonstrated the ability to transform tetrachloro- to octachlorodibenzo-p-dioxins into less chlorinated dioxins, including monochlorinated congeners [31]. Bacterial community investigation carried out by Dam et al. [32] revealed that the *Dehalococcoides mccartyi* strain was involved in the reductive dechlorination of 1,2,3,4-TeCDD.

**Figure 6:**
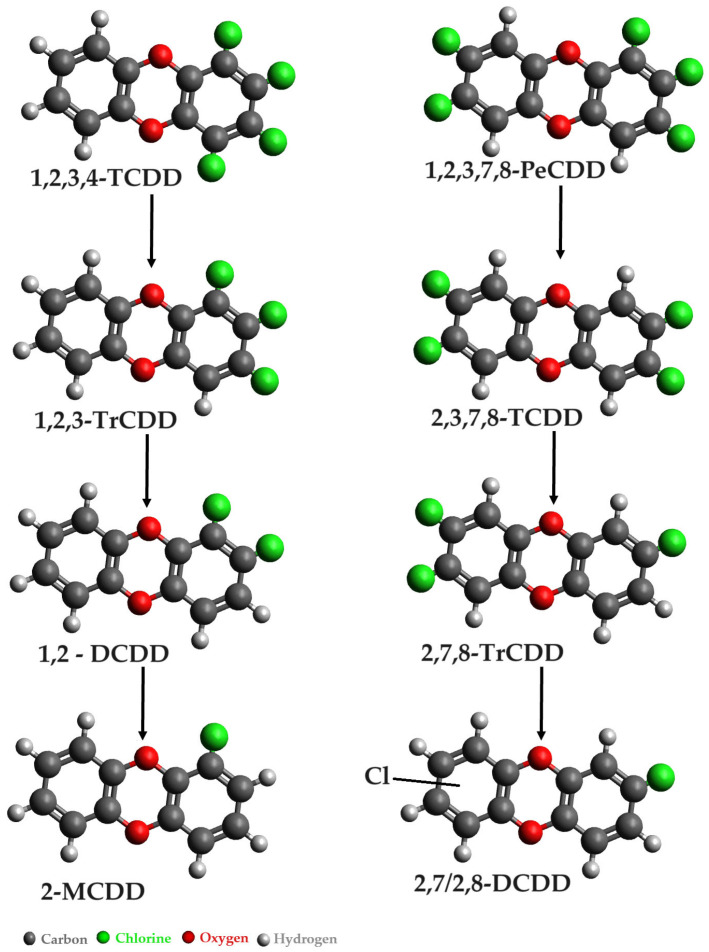
Anaerobic degradation of polychlorinated dioxins by *Dehalococcoides* sp. [5].

The potential pathways for the dechlorination of 1,2,3,4-TCDD and 1,2,3,7,8-PeCDD are depicted in Figure 6. These pathways represent intricate sequences of chemical transformations, illustrating the stepwise removal of chlorine atoms from the dioxin structures and shedding light on the complex mechanisms involved in the degradation of these dioxins [5].

Bunge and Lechner’s study [33] revealed the reductive dechlorination capabilities of three anaerobic strains, specifically *Dehalococcoides mccartyi* strain 195, CBDB1, and DCMB5, in relation to polychlorinated dibenzo-p-dioxins (PCDDs). Notably, *Dehalococcoides ethenogenes* strain 195, known for its proficiency in dechlorinating tetrachloroethene to vinyl chloride and ethene, exhibited the ability to dechlorinate 1,2,3,4-TCDD and 1,2,3,4-TCDF [34,35].

Furthermore, other bacterial strains such as *Sphingomonas wittichii* RW1, *Pseudomonas* sp. strain HH69, and *Burkholderia* sp. JB1 have been identified for their capacity to degrade higher chlorinated dioxins and furans. It is worth noting, however, that these strains were unable to utilize these congeners as a carbon and energy source for growth. Despite the existence of bacterial species capable of degrading certain dioxins and furans, their efficacy is limited, with proven capabilities restricted mainly to monochlorinated congeners and some isomers of di- and tri-chlorinated congeners. Unfortunately, the most toxic congener, 2,3,7,8-tetrachlorodioxin, along with other dioxins and polychlorinated furans, has been found to be practically resistant to this decomposition pathway [36].

### 3.2. Fungus-Assisted Biodegradation of POPs from Wastewaters

Fungi, as eukaryotic organisms, exhibit the ability to thrive on diverse substrates and have an enduring lifespan. They have demonstrated remarkable efficacy in the biodegradation of waste, wastewater remediation, and the breakdown of various materials including wood, textiles, plastics, and items contaminated with hazardous substances such as organochlorine pesticides, PCB-based electrical insulating oils, and polycyclic aromatic hydrocarbons. This capability is attributed to the utilization of specific enzymes and involves oxidative and reductive mechanisms [1,37].

**Figure 7:**
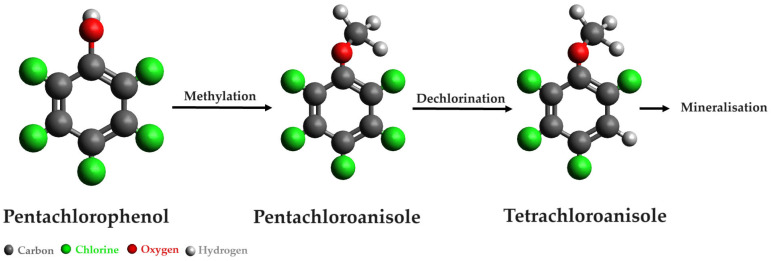
Pentachlorophenol biodegradation mechanism [38].

Numerous studies have explored the fungal degradation of halogenated hydrocarbons, with a focus on pentachlorophenol (PCP), intensively used as an insecticide, fungicide, herbicide, and wood preservative, and used in the chlorination of wastewater. In a study by Vacondio et al. [38], five fungal strains from the species *Trichoderma* sp., *Aspergillus* sp., *Cladosporium* sp., and *Fusarium* sp. were employed for the mycodegradation of PCP. Remarkably, *Trichoderma harzianum* CBMAI 1677 exhibited outstanding performance, completely degrading PCP within a week of incubation. Furthermore, this fungal strain demonstrated effectiveness in the biodegradation of penta- and tetra-chloroanisole. A likely PCP biodegradation mechanism by the strain *Tricoderma harzianum* is illustrated in Figure 7.

Birolli et al. [39] screened some marine-derived fungi (*Trichoderma harzianum* CBMAI 1677, *Cladosporium* sp. CBMAI 1237, *Aspergillus sydowii* CBMAI 935, *Penicillium citrinum* CBMAI 1186 and *Mucor racemosus* CBMAI 847) for anthracene biodegradation. They noticed an increase in mycelium weight, presuming that anthracene may act as a carbon source for the investigated microbes. In the same study, *Cladosporium* sp. CBMAI 1237 was also tested for the biodegradation of other polycyclic aromatic hydrocarbons such as acenaphthene, fluorene, phenanthrene, fluoranthene, pyrene and nitropyrene, and the biodegradation level exceeded 50%.

In their study, Dao et al. [40] showcased the effectiveness of the ligninolytic fungus *Rigidoporus* sp. FMD21 and its extracellular enzymes in degrading of 2,3,7,8-TCDD under mild conditions. The biotransformation process was notably influenced by the pivotal role of laccases throughout the degradation. The application of extracellular enzymes facilitated the degradation of 2,3,7,8-TCDD, resulting in the production of 3,4-dichlorophenol (3,4-DCP) as the primary intermediate product.

Zhou et al. [41] used the fungus *Phanerochaete chrysosporium* to remove the perfluorooctanoic acid (PFOA) in a liquid culture system. Initially, the presence of perfluorooctanoic acid led to the inhibition of enzyme activities in the *Phanerochaete chrysosporium* incubation system. However, after a 14-day incubation period, enzyme activities were subsequently enhanced. The maximum removal efficiency for PFOA, reaching 69.23%, was observed after 35 days in the *P. chrysosporium* incubation system, starting with an initial PFOA concentration of 0.002 mM.

Bumpus et al. [42] demonstrated that white rot *Planerochaete chrysosporium* could mineralize 2,3,7,8-TCDD and other aromatic compounds, at different mineralization degrees. The peroxidase enzyme secreted by this fungus is responsible for the oxidation of many aromatic compounds, including chlorinated dioxins [36]. Also, fungi belonging to the genera *Aleurodiscus*, *Ceriporia*, *Phanerochaete* and *Phlebia* were found to be able to degrade dioxins in a complex study where 166 strains of wood rot fungi were employed [43].

Derived from contaminated soil, a *Penicillium* sp. strain exhibited notable proficiency in degrading 2,3,7,8-TCDD from wastewater [44]. Experimental findings elucidated the formation of intermediates during the degradation of 2,3,7,8-TCDD, encompassing benzoquinone, catechol, hydroxybenzene, and β-ketoadipic acid, the latter being found as a key component in the degradation pathway of 2,3,7,8-TCDD.

A method for the degradation of dioxins and furans by white rot fungus *Phanerochaete sordida* YK-624 was developed by Takada et al. [45]. The fungus *P. sordida* YK-624 degraded not only PCDDs and PCDFs but also tetra- to octa-chloro substituents.

*Pleurotus pulmonarius* LBM 105 exhibited a remarkable capacity to remove more than 95% of polychlorinated biphenyls (PCBs) within 24 days in a nitrogen-limited mineral medium, in a study performed by Benitez et al. [46]. In the same study, the percentages of PCB removal by *T. sanguinea* LBM 023 was about 59%, while in that in a binary consortium was only 47%.

## 4. Conclusions, Challenges and Future Perspectives

Persistent organic pollutants (POPs) are toxic chemicals that pose a significant threat to the environment and human health due to their persistence, bioaccumulation, and adverse effects. These compounds can enter the water ecosystem through various sources, including industrial discharges and domestic wastewater. Traditional water treatment methods often struggle to effectively remove POPs, requiring the development of alternative strategies.

Bacteria and fungi play an important and irreplaceable role in the removal of persistent organic pollutants (POPs) from wastewater, offering a natural and sustainable solution to address environmental contamination challenges. Their unique metabolic capabilities allow them to enzymatically degrade a wide range of complex organic compounds, including hydrophobic POPs that are otherwise challenging to eliminate through conventional treatment methods.

POP removal via biological methods is an approach that still faces some limitations and challenges, among which are the longer decontamination time, the sensitivity of microbial species to the toxicity of the pollutant, the dynamics of bioremediation processes, and the complexity in controlling the dynamic processes dictated by microorganisms. The ability of bacteria and fungi to adapt to diverse environmental conditions makes them versatile agents for pollutant removal, while their synergistic interactions in microbial consortia further enhance their efficiency.

Wastewater, a complex matrix laden with diverse pollutants, presents a formidable challenge in eliminating persistent organic pollutants (POPs). The intricate nature of these pollutants, often hydrophobic and prone to forming complexes with organic matter, hinders their accessibility for microbial degradation. Moreover, some POPs exhibit toxicity to microorganisms, while co-contaminants and antimicrobial agents in wastewater can further impede bacterial and fungal activity.

It is noteworthy that the vast majority of environmental organisms cannot be isolated and grown in axenic culture. This presents a significant challenge in the study and application of biodegradation processes. Many microorganisms involved in the degradation of persistent organic pollutants (POPs) are part of complex microbial communities that rely on interactions with other organisms to survive and function effectively. These symbiotic relationships are often difficult to replicate in laboratory settings, where axenic cultures are required. Consequently, the inability to culture these organisms in isolation hinders the identification and utilization of potentially effective biodegraders, limiting advancements in bioremediation technologies.

Moreover, the use of microorganisms in treatment processes can pose various health risks, such as infections or the release of harmful byproducts, if not properly controlled. To mitigate these risks, it is important to implement stringent safety protocols, including regular monitoring of microbial activity, maintaining appropriate environmental conditions to prevent the proliferation of pathogenic strains or using different methods of disinfection (e.g., chemical disinfectants, antimicrobials or UV-exposure) before water disposal in the environment.

Important progress has been made in the direction of the bioremediation of POP-contaminated wastewater, but there are still many issues that require more in-depth investigation, so that this approach becomes feasible and applicable in large-scale projects. Investigating the species of bacteria and fungi capable of metabolizing these chemicals at a sufficiently high level of biodegradability compared to conventional strategies is necessary. In addition, the metabolic engineering of new microbial strains and encoding genes for the development of selective species in the biodegradation of POPs could be a future approach in fundamental research. Also, research on the integration of microbiological bioremediation techniques with electrophysical techniques such as the use of ionizing radiation could bring benefits both in terms of increasing the degree of biodegradation and in terms of reducing the processing time. The development of high-performance biocatalysts secreted by microorganisms can help increase the performance of the biochemical processes of transforming toxic substances into harmless or less toxic compounds. An exploitation direction that would bring not only environmental benefits but also important economic benefits is conducting research to reduce the operating costs of water treatment plants and efficient sludge management. Since most laboratory research is carried out in batch experiments, it is important to also approach experiments in continuous flow processes to facilitate the efficient exploitation of these results in industrial applications. The results of this study can help to provide up-to-date theoretical information to students and researchers who want to deepen this scientific field, as well as to developers of treatment plants who want to invest in innovative, cost-effective and ecofriendly methods for the bioremediation of POP-contaminated wastewaters.
